# Free Amino Acids in Three *Pleurotus* Species Cultivated on Agricultural and Agro-Industrial By-Products

**DOI:** 10.3390/molecules25174015

**Published:** 2020-09-02

**Authors:** Dimitra Tagkouli, Andriana Kaliora, Georgios Bekiaris, Georgios Koutrotsios, Margarita Christea, Georgios I. Zervakis, Nick Kalogeropoulos

**Affiliations:** 1 Department of Dietetics-Nutrition, School of Health Science and Education, Harokopio University of Athens, El. Venizelou 70, Kallithea, 17676 Athens, Greece; dtagkoul@hua.gr (D.T.); akaliora@hua.gr (A.K.); mchristea@hua.gr (M.C.); 2 Laboratory of General and Agricultural Microbiology, Agricultural University of Athens, Iera Odos 75, 11855 Athens, Greece; giorgosbekiaris@yahoo.gr (G.B.); georgioskoutrotsios@gmail.com (G.K.)

**Keywords:** *Pleurotus* mushrooms, free amino acids, GABA, bitter amino acids, sweet amino acids, monosodium glutamate-like amino acids, wheat straw, grape marc, olive mill waste, PCA

## Abstract

Previous studies have demonstrated the feasibility of employing by-products of the olive and wine sectors for the production of *Pleurotus* mushrooms with enhanced functionalities. In this work we investigated the influence of endogenous and exogenous factors on free amino acids (FAAs) profile of *Pleurotus ostreatus*, *P. eryngii* and *P. nebrodensis* mushrooms produced on wheat straw (WS), alone or mixed with grape marc (GM), and on by-products of the olive industry (OL). Overall, 22 FAAs were determined in substrates and mushrooms, including all the essential amino acids, the neurotransmitter γ-aminobutyric acid (GABA) and ornithine. On a dry weight (dw) basis, total FAAs ranged from 17.37 mg/g in *P. nebrodensis* to 130.12 mg/g in *P. ostreatus* samples, with alanine, leucine, glutamine, valine and serine predominating. Similar distribution patterns were followed by the monosodium glutamate (MSG)-like, sweet and bitter FAAs. Significant differences in FAAs level were observed among the species examined and among the cultivation substrates used. Principal Component Analysis (PCA) performed on the entire FAAs profile of six *Pleurotus* strains, clearly separated *P. ostreatus* from *P. eryngii* and *P. nebrodensis*, in accordance to their phylogenetic affinity. This is the first report of FAAs in *P. nebrodensis.*

## 1. Introduction

Edible mushrooms are considered delicacies due to their distinct texture, aroma and exceptional flavor. In addition, they exhibit several health-beneficial effects, e.g., hypocholesterolaemic, antihypertensive, cardioprotective and chemopreventive [1,2,3]. The world production of cultivated edible mushrooms considerably increased during the last 20 years and has reached 34 billion kg in 2013, with *Pleurotus* representing circa 19% of the total [4]. Amongst the *Pleurotus* species, *P. ostreatus* is the most widely cultivated, while *P. eryngii* and *P. nebrodensis* produce mushrooms of exceptional organoleptic properties [5]. Since *Pleurotus* fungi are efficient in biodegradation of a wide range of lignocellulosic residues and by-products, including those originating from the olive oil and wine sector agro-industries [6,7], their exploitation as mushroom cultivation substrates is of great applied interest. We have previously shown that such materials can be successfully used for the production of value-added biomass, i.e., mushrooms enriched with bioactive compounds and enhanced antioxidant activity [8,9,10].

Amino acids (AAs) not only act as substrates for protein synthesis, but they are also associated with significant health-related effects by being involved in various pathways of cellular communication, gene expression, oxidative stress, immune process and intracellular protein metabolism [11]. In addition, free amino acids (FAAs) together with other non-volatile compounds, such as 5′-nucleotides, soluble sugars and polyols, are considered responsible for the palatable taste of mushrooms [12,13,14,15,16]. As such, the identification of the AAs profile in foods is of particular importance from an organoleptic and physiological point of view, while it is crucial in metabolic research studies and in a wide range of medical and biopharmaceutical applications. Especially in edible mushrooms that are complex matrices rich in beta-glucans, minerals, vitamins and bioactive compounds (i.e., ergosterol, tocopherols, phenolics, organic acids, lovastatin, etc.) [8,17,18], the simultaneous presence of all essential AAs, gamma-aminobutyric acid (GABA) and ornithine further confers to their high nutritional value.

Hereby, we sought to assess the FAAs profile in *P. ostreatus*, *P. eryngii* and *P. nebrodensis* mushrooms, and to investigate how FAAs are influenced by endogenous (through the examination of six *Pleurotus* strains) and exogenous (by including fruitbodies produced in various cultivation substrates) factors. 

## 2. Results and Discussion

Overall, 22 FAAs were detected and quantified in mushroom samples of six *Pleurotus* strains and in their cultivation substrates. 

The FAAs were determined by the EZ:faast™ kit for GC-MS provided by Phenomenex (Torrance, CA, USA) [19]. The aforementioned kit can measure all the essential and several non-essential AAs, whereas it does not measure the conditionally essential cysteine and arginine, together with citrulline, 1- and 3-methylhistidines and taurine [20].

Depending on their properties, AAs are placed in several groups that portray their essential role in human diet not only in terms of nutritional importance but also as regards their contribution to food palatability. Such groups are: essential AAs that cannot be synthesized by the human body and must be obtained from food sources, umami-taste active or monosodium glutamate (MSG)-like AAs, branched-chain AAs (BCAAs), bitter-taste AAs and sweet-taste AAs (Table 1).

### 2.1. Protein and FAAs Content in Mushroom Cultivation Substrates

The total content of FAAs detected in mushroom cultivation substrates ranged from 0.33 mg/g dw in WS to 0.71 mg/g dw in OL and 1.23 mg/g dw in GM (Table 2). FAAs profiles exhibited notable differences among substrates, with glutamic acid, glutamine and lysine being the most abundant in WS, representing 69% of total FAAs; glutamic acid, asparagine, thioproline, aspartic acid, glutamine and proline dominated in GM (circa 60% of total FAAs) and thioproline, aspartic acid, tryptophan, asparagine and glutamic acid comprised 71% of total FAAs in OL. Crude protein content of substrates was found equal to 85.2, 116.6 and 129.0 mg/g dw in WS, GM and OL, respectively. Considering that the GM substrate is composed by equal weights of wheat straw and grape marc, while the OL substrate is composed by olive leaves and two-phase olive mill wastes at 3:1 *w*/*w* ratio, the protein content of substrates determined in the present work is in agreement to data provided in the literature for the protein content of substrate components, which increase in the order: olive mill wastes (4 mg/g dw) < wheat straw (43 mg/g dw) < grape pomace (110–160 mg/g dw) < olive leaves (196 mg/g dw) [21,22].

### 2.2. FAAs Profiles in Mushrooms

The FAA content in *Pleurotus* mushrooms is presented in Table 3. On a dry weight basis, the most abundant FAAs in *P. ostreatus* were glutamine (7.8–21.69 mg/g), followed by leucine (8.59–16.45 mg/g), alanine (7.22–13.33 mg/g), valine (4.24–9.49 mg/g), glutamic acid (4.81–7.84 mg/g) and serine (3.93–8.83 mg/g). The same FAAs, but in a different order, predominated in *P. eryngii*, i.e., leucine (5.11–11.24 mg/g), followed by alanine (4.74–8.54 mg/g), glutamine (3.35–8.28 mg/g), valine (3.24–6.24 mg/g), serine (3.30–5.97 mg/g) and glutamic acid (3.22–5.73 mg/g). Finally, leucine (2.24–8.29 mg/g) and alanine (2.82–7.53 mg/g) were the most abundant FAAs in *P. nebrodensis*, followed by glutamine (0.84–7.02 mg/g), glutamic acid (2.07–4.59 mg/g), serine (0.91–4.19 mg/g) and valine (1.05–3.98 mg/g). Among the species studied, *P. nebrodensis* presented the lower concentrations in most FAAs.

In literature, glutamic acid, alanine, arginine and threonine have been reported among the predominating FAAs in *Pleurotus* species cultivated on several lignocellulosic wastes, followed in decreasing frequency by leucine, aspartic acid, valine and serine [12,15,23]. The same group of amino acids, with glutamic acid present in higher amounts, also predominated in other mushroom species, such as *Agaricus brasiliensis, Lentinula edodes, Hericium erinaceus, Cordyceps militaris, Grifola frondosa, Coprinus comatus, Flammulina velutipes* and *Tremella fuciformis* [24]. Glutamic acid, followed by alanine and aspartic acid predominated also in *Agaricus bisporus* and *Lentinula edodes* samples [25]. Deviations from the findings of the present study can be explained by differences in the composition of the substrates used and by the species/strain examined.

#### 2.2.1. Protein and Total FAAs Content

Total FAAs and protein content of the mushrooms studied are shown in Table 3. Crude protein content ranged from 121.1 mg/g dw in *P. nebrodensis* UPA 6 grown on WS to 293.2 and 299.2 mg/g dw in *P. ostreatus* LGAM 11 cultivated on OL and GM, respectively. On average, the crude protein content in *P. ostreatus* mushrooms was notably higher than in *P. eryngii* and *P. nebrodensis* fruitbodies (Table 3). With regard to substrates, *P. ostreatus* mushrooms cultivated on OL presented the highest protein content, followed by those grown on GM and WS. The other two species studied presented a different pattern, with their protein values decreasing in the order GM > OL > WS.

Total FAAs content ranged from 17.37 mg/g dw in *P. nebrodensis* UPA 6 produced on WS to 130.12 mg/g dw in *P. ostreatus* LGAM 11 cultivated on GM, following similar patterns of distribution to those of crude protein. Total FAAs corresponded (in average) to 41.6, 37.1 and 28.3% of total protein in *P. ostreatus*, *P. eryngii* and *P. nebrodensis*, respectively; the respective fractions were 34.2, 37.7 and 35.1% for mushrooms produced on WS, GM and OL (Table 3). Among the species studied, *P. ostreatus* exhibited the highest FAAs content, followed by *P. eryngii* and *P. nebrodensis* regardless of the substrate used (Table 3). The outcome of this study is in agreement with previously reported results evidencing that *P. ostreatus* mushrooms present higher FAAs content compared to *P. eryngii* [25,26].

**Figure 1:**
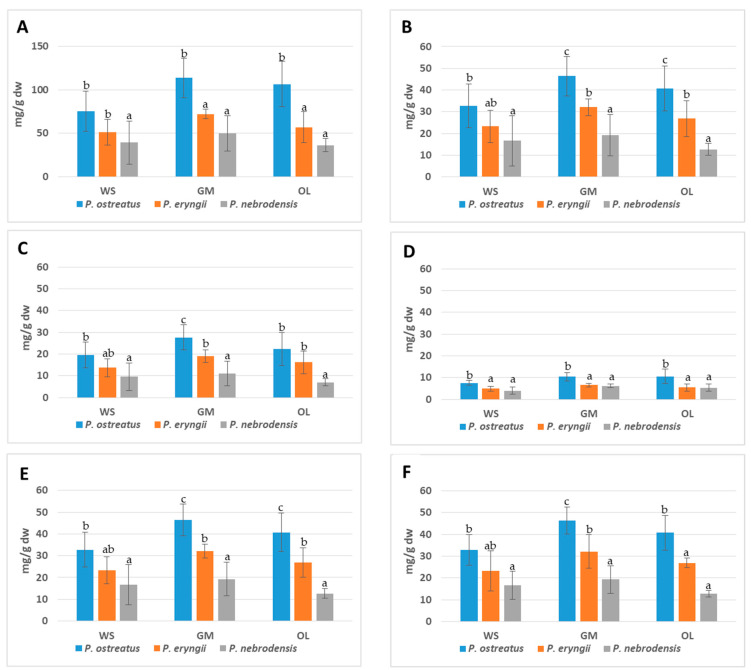
Total and individual groups of free amino acids in *Pleurotus* mushrooms, compared by species in each substrate. (**A**) Total Amino Acids, (**B**) Essential Amino Acids, (**C**) Branched Chain Amino Acids; (**D**) Monosodium Glutamate (MSG)-like Amino Acids, (**E**) Bitter Taste Amino Acids, (**F**) Sweet Taste Amino Acids. Lack of letters in common denotes statistically significant differences in comparisons among species in each substrate by Duncan’s multiple comparison test at *p* < 0.05. Essential amino acids, Thr + Val + Met + Ile + Leu + Phe + Lys + His+ Trp; BCAA Branched chain amino acids, Val + Ile + Leu; MSG-like, monosodium glutamate-like, Asp + Glu; Bitter, Val + Met + Ile + Leu + Phe + His + Trp; Sweet, Thr + Ser + Gly + Ala + Pro.

Regarding the effect of cultivation substrate on mushrooms’ FAAs content, the use of GM substrate resulted in a statistically significant increase of FAAs content in the produced mushrooms when compared to the control (WS) for all the species studied, while in the case of cultivation on OL, differences were observed only in *P. ostreatus* (Figure 1). Therefore, it appears that the use of GM in *Pleurotus* cultivation substrates leads to enhanced FAAs production. The addition of olive mill wastes (OL), although resulting in increased FAAs content, inhibited the growth of certain *Pleurotus* strains, as has been previously shown [8]. 

Previous studies on total FAAs in *Pleurotus* mushrooms exhibit wide fluctuations, with reported values ranging from 3.3 to 192 mg/g dw [12,15,23,26,27,28]. Large variations of total FAAs values have been also reported for other widely cultivated mushroom species, e.g., 10.1–55.7 mg/g dw for *L. edodes* [15,24,25,26,29], 18.8–168.4 mg/g dw for *A. bisporus* [24,25,26] and 19.2–147.1 mg/g dw for *F. velutipes* [15,24,25,26].

Such fluctuations are not surprising given that, besides the influence of genetic factors (i.e., species and strains), mushrooms composition is additionally affected by the stage of development, the nature of pre- and post-harvest treatments and the type of growth substrate [30]. Additionally, different methods of FAAs isolation and quantification may result in such inconsistencies. The values of FAAs determined in the present work are among the highest reported for *Pleurotus* mushrooms.

#### 2.2.2. Essential FAAs

Mushrooms usually contain all the essential FAAs, which comprise 25–40% of total FAAs content [31]. In the present study, all essential FAAs were detected in the samples examined, representing in average the 41, 46 and 38% of total FAAs in *P. ostreatus, P. eryngii* and *P. nebrodensis* mushrooms, respectively; their relative contents were significantly higher than those measured in the cultivation substrates, i.e., 19, 22 and 22% of total FAAs in WS, GM and OL, respectively (Table 2). The essential FAAs followed similar distribution patterns to those of total FAAs, and hence, the highest values were noted in *P. ostreatus* mushrooms (39.13 and 53.33 mg/g in WS and GM substrates by LGAM 11, and 43.08 mg/g in OL by LGAM 14) (Supplementary Table S2), whereas the lowest concentrations were recorded in fruitbodies of *P. nebrodensis* UPA 6 (6.43 to 13.13 mg/g dw). Literature data for essential FAAs in cultivated *Pleurotus* mushrooms presented wide fluctuations with reported values ranging from 1.76 mg/g in *P. eryngii* to 78.2 mg/g dw in *P. ostreatus* [12,15,23,32,33,34]. Among the essential FAAs of the *Pleurotus* species and strains studied, leucine predominated, followed by valine, isoleucine, threonine, phenylalanine and lysine (Table 2). In agreement with our findings, leucine together with valine and lysine were reported to predominate among the essential FAAs in three *Pleurotus* species grown on wheat stalk [32], and in *P. ostreatus* grown on several lignocellulosic agro wastes [35]. When poplar sawdust with rice bran was used as cultivation substrate, threonine predominated among the essential FAAs in five out of six *Pleurotus* species, with leucine predominating only in *P. djamor* [28]. In addition, studies on total AAs also reported leucine to be the most abundant essential amino acid in *P. ostreatus* powder [36], in wild and cultivated “P. sajor-caju” [37], in seven strains of *P. ostreatus* grown on wheat straw with sugar beet [30] and in three out of four commercial *Pleurotus* species [27]. 

#### 2.2.3. Branched Chain Amino Acids, GABA and Ornithine

BCAAs are necessary for the synthesis of proteins and consequently for the production of immunoglobulins, cytokines and their receptors. Thus, BCAAs availability in the diet is of importance since they also exhibit a significant immunoregulating role [38], which is already well established for mushrooms beta-glucans [39,40]. In the present study the highest concentrations of free BCAAs were recorded in *P. ostreatus* fruitbodies, followed by *P. eryngii* and *P. nebrodensis*; *P. ostreatus* LGAM 11 cultivated on GM demonstrated the highest value (31.73 mg/g dw), while *P. ostreatus* LGAM 14 cultivated on OL produced mushrooms with higher amounts of BCAAs (23.19 mg/g dw) than those grown on WS (15.64 mg/g dw). The lowest BCAAs contents were observed in *P. nebrodensis* UPA 6 (3.96 to 7.52 mg/g dw). Among substrates, cultivation on GM resulted in the higher amounts of leucine, valine and isoleucine in *P. eryngii* UPA 12 and LGAM 212 and in *P. nebrodensis* UPA 6; *P. ostreatus* LGAM 11 cultivated on GM and *P. ostreatus* LGAM 14 cultivated on OL produced mushrooms with the higher valine and isoleucine contents. Values for free BCAAs content obtained herein fall within the range of 0.64–36.75 mg/g dw reported in the literature for several *Pleurotus* species [28,32,33,34,35,41]. Regarding individual BCAAs, leucine predominated in all cases of the present study, followed by valine and isoleucine. The outcome of previous publications [32,35] are in line with our findings including reports with valine predominating over leucine [33,34,41]. On the other hand, Yin et al. [28] showed that leucine content was higher than valine’s in four out of six *Pleurotus* species.

GABA and ornithine are two non-essential AAs with significant physiological activities. GABA is a non-protein, four carbon AA present in plants, animals and microorganisms. It participates in the Krebs cycle, and behaves as a potent neurotransmitter in the central nervous system of vertebrates by decreasing neuron activity [42]. Ornithine is a precursor in the synthesis of arginine and participates in the urea cycle. It is involved in the production of excess growth hormone and in the burn up of excess fat in the body. Moreover, ornithine’s role in the immune system and liver functions is considered crucial [43]. 

In the present work, low amounts of GABA were measured in all mushroom and substrate samples; GABA was not detected in the WS substrate (Table 2). In mushrooms cultivated on WS, free GABA concentrations ranged from 0.08 mg/g dw in *P. eryngii* UPA 12 to 0.56 mg/g dw in *P. nebrodensis* LGAM 162. In mushrooms produced on GM, GABA concentrations varied between 0.18–0.86 mg/g dw, with the lowest observed in *P. eryngii* LGAM 212 and *P. nebrodensis* UPA 6, and the highest in *P. nebrodensis* LGAM 162. Finally, the GABA content of mushrooms cultivated on OL ranged from 0.06 to 0.46 mg/g dw in *P. eryngii* UPA 12 and *P. ostreatus* LGAM 14, respectively. Lower GABA concentrations were observed in mushrooms grown on WS compared to the other substrates, the decrement in most cases being not statistically significant. Overall, GABA concentrations ranged from 0.06 to 0.86 mg/g dw (Table 3). Previous studies on the GABA content in *Pleurotus* mushrooms reported a high variability with values ranging from 0.06 to 3.91 mg/g dw [26,28,30,33,34], while the respective content in mycelia of 13 *Pleurotus* species was reported to range from 0.20 mg/g dw in “P. florida” to 2.81 mg/g dw in *P. eryngii* [44]. 

Recently, Park et al. [45] examined GABA production by adding selected AAs in liquid cultures of *L. edodes*, and observed a three-fold increase of GABA in respect to the control when the medium was supplemented with glutamic acid, whereas supplementation with alanine and glycine did not affect GABA production. The findings of this study are consistent with the fact that GABA is formed by the decarboxylation of glutamic acid or its salts [42,46]. In the substrates used in the present study, glutamic acid predominated among FAAs in WS and GM at concentrations equal to 0.155 and 0.200 mg/g dw, respectively, while it was lower in OL (0.064 mg/g dw; Table 2). Substrates content in free glutamic acid and crude protein (lower in WS compared to GM and OL) could partly explain the increased GABA concentrations measured in mushrooms of most strains cultivated on GM in respect to those cultivated on WS (Table 3). 

Free ornithine was also detected in all mushroom and substrate samples at concentrations ranging from 0.36 to 1.51 mg/g dw in *P. eryngii* LGAM 212 and *P. nebrodensis* LGAM 162 (cultivated on WS), respectively, from 0.50 to 1.63 mg/g dw in *P. eryngii* LGAM 212 and *P. nebrodensis* LGAM 162 (cultivated on GM), respectively, and from 0.31 to 1.10 mg/g dw in *P. eryngii* UPA 12 and *P. ostreatus* LGAM 14 (cultivated on OL), respectively. Overall, ornithine concentrations in the present study did not exhibit any specific trend and/or correlation with substrates or species/strains. Free ornithine values of 0.23–3.80 for *P. ostreatus* and 1.40–8.45 mg/g dw for *P. eryngii* have been previously reported in mushrooms cultivated on wheat straw, poplar sawdust and rice bran, and on sugar beet with wheat straw [26,28,30].

#### 2.2.4. Taste Attributes

In regard to the palatability characteristics of mushrooms studied, *P. ostreatus* exhibited the highest concentration of bitter taste FAAs, with strain LGAM 11 cultivated on WS and GM reaching 31.95 and 43.39 mg/g dw and strain LGAM 14 cultivated on OL reaching 33.65 mg/g dw. The lower amounts of bitter AA were measured in *P. nebrodensis* UPA 6, i.e., 5.29 to 10.59 mg/g dw (Supplementary Table S2). In the literature, bitter FAAs content for *P. ostreatus* and *P. eryngii* were reported to range from 0.78 to 51 mg/g dw and from 2.7 to 37 mg/g dw, respectively [12,15,23,28,32].

The distribution pattern of sweet FAAs was similar to that of bitter FAAs, with higher values obtained in *P. ostreatus* LGAM 11 cultivated on WS and GM (24.15 and 34.56 mg/g dw, respectively) and *P. ostreatus* LGAM 14 grown on OL (33.28 mg/g dw). Lower amounts of sweet FAAs were recorded in *P. nebrodensis* UPA 6, i.e., 5.19 to 8.74 7.82 mg/g dw (Supplementary Table S2). Sweet AAs previously reported for *P. ostreatus* ranged from 0.77 mg/g dw in mushrooms cultivated on wheat and paddy straw [35], and 12.3 mg/g dw in fruitbodies obtained on poplar sawdust supplemented with rice bran [28], up to 48 mg/g dw in mushrooms cultivated on wheat stalks [32], or 13.8 mg/g dw in specimens collected in nature [41]. The respective literature data for sweet FAAs in *P. eryngii* mushrooms range from 1.7 to 35 mg/g dw in mushrooms cultivated in commercial substrates and on wheat stalks, respectively [23,32].

Among the AAs determined, aspartic acid and glutamic acid are known to contribute to the umami taste of foodstuffs, and are characterized as monosodium glutamate-like (MSG-like) AAs [29,47,48]. In the present study the highest concentrations of MSG-like FAAs were recorded in *P. ostreatus* and the lowest in *P. nebrodensis* samples. Mushrooms of both *P. ostreatus* strains cultivated on GM and OL contained more than 10 mg/g dw of MSG-like FAAs (Figure 1; Supplementary Table S2). The lower amounts of MSG-like FAAs were observed in *P. nebrodensis* UPA 6 fruitbodies grown on WS, GM and OL (2.83, 6.18 and 5.40 mg/g dw, respectively). Mau et al. [16] ranked edible and medicinal mushrooms according to their equivalent umami concentrations and observed that, among the *Pleurotus* species, *P. citrinopileatus* exhibited the highest values followed by *P. eryngii* and *P. ostreatus*. Moreover, Mau et al. [15], classified mushrooms according to their MSG-like FAAs contents as high (>20 mg/g), middle (5–20 mg/g) and low (<5 mg/g). Hence, the mushrooms investigated hereby are classified as middle (4.2 to 10.9 mg/g dw), except for the *P. eryngii* UPA 12 fruitbodies cultivated on WS and OL, which are classified as low. Our results are in agreement with those reported in literature for MSG-like FAAs content in *Pleurotus* mushrooms, i.e., 0.84–48 mg/g dw [12,15,23,28,33,34].

### 2.3. Principal Component Analysis 

**Figure 2:**
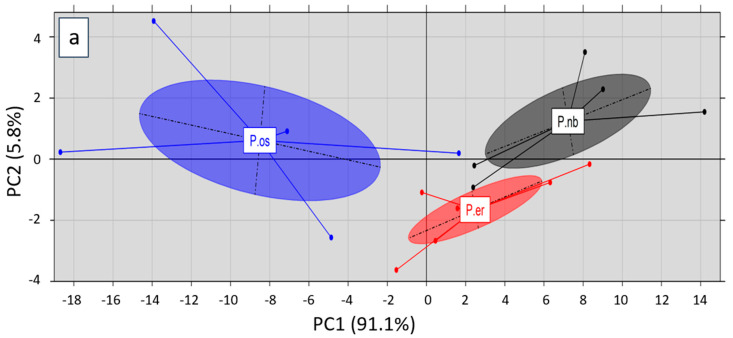

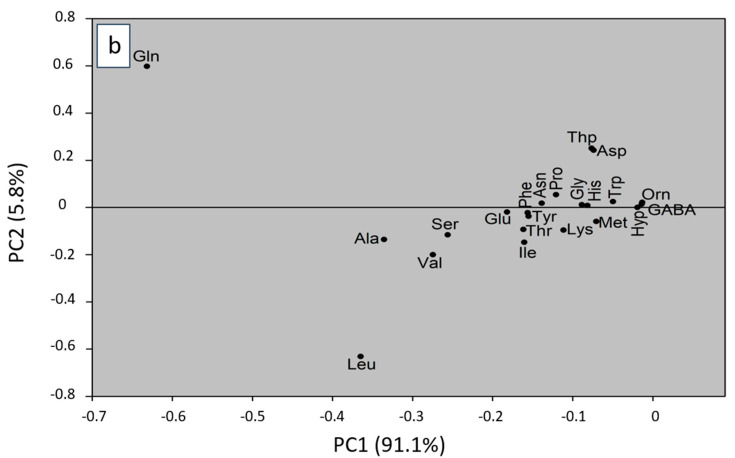
(**a**) Score plot of PCA (PC1 vs. PC2) for the discrimination among *Pleurotus* species (P. os, *P. ostreatus*; P. er, *P. eryngii*; P. nb, *P. nebrodensis*); (**b**) Plot with the loadings of the amino acids related to the discrimination among *Pleurotus* species.

A Principal Component Analysis (PCA) was performed on the entire FAAs profile of the three *Pleurotus* species in order to investigate the existence of any groupings or associations. The first two principal components explained most of the data set variance (i.e., 96.9%), while after the projection of the first (PC1; 91.1%) against the second principal component (PC2; 5.8%), three independent clusters were formed corresponding to the data sets of *P. ostreatus, P. eryngii* and *P. nebrodensis* (with ellipses drawn at a confidence level of 0.95). The difference on ellipses’ size indicated variations among the determined total FAAs content for each species cultivated on the three substrates. *P. ostreatus* was distinctly separated from *P. eryngii* and *P. nebrodensis* across PC1 (*x*-axis), in accordance with their phylogenetic affinity [5]. Moreover, *P. eryngii* was distinguished from *P. nebrodensis* across PC2 (*y*-axis) (Figure 2a). The observation of the responsible loadings (Figure 2b) revealed an impact of both PCs on the grouping, particularly as regards PC1. Amino acids such as glutamine, leucine and alanine seem to dominate *P. ostreatus* strains, while methionine, proline, tryptophan, isoleucine and others appear to be related to *P. eryngii* and *P. nebrodensis*. On the other hand, the observation of PC2 loadings revealed a positive correlation of *P. nebrodensis* species with AAs such as glutamine, threonine, aspartic acid and proline. A positive correlation of *P. eryngii* species with AAs such as leucine, alanine, serine and valine was also observed across the PC2 loadings (negative values). When the analysis was conducted on the basis of the cultivation substrates used, no grouping of mushrooms was observed (Figure S1, Supplementary Material). This indicates a significant effect of different *Pleurotus* species on the FAAs profiles, whereas the effect of different substrates was not discernible. Recently, the application of PCA on FAAs and 5′-nucleotides of shiitake mushrooms was found suitable for the discrimination of species and geographical origins [49]. 

## 3. Materials and Methods 

### 3.1. Biological Material and Mushroom Cultivation Substrates

*P. ostreatus* strains LGAM 11 and LGAM 14, *P. eryngii* strains UPA 12 and LGAM 212 and *P. nebrodensis* strains UPA 6 and LGAM 162 were examined. Biological material is maintained in the Culture Collection of the Agricultural University of Athens (Laboratory of General and Agricultural Microbiology, Athens, Greece). Three mushroom cultivation substrates were used consisting of wheat straw (WS) as control, wheat straw in 1:1 *w*/*w* ratio with grape marc (GM), and olive leaves in 3:1 *w*/*w* ratio with two-phase olive mill wastes (OL). WS were obtained from the Thessaly region (central Greece), GM from a winery in the Nemea area (northeast Peloponnese, Greece) and OL from an olive-oil mill in Kalamata (southwest Peloponnese, Greece). Spawn and substrate preparation methodologies as well as mushroom cultivation conditions were as previously described [8,9].

### 3.2. Sample Pre-Treatment

Mushroom samples (in triplicates) from each treatment were freeze-dried (HetoLyolab3000, Heto-Holten, Allerød, Denmark), pulverized, placed in plastic bags and kept at −40 °C until analysis. The same procedure was followed for substrate samples.

### 3.3. Extraction of Free Amino Acids

Free amino acids were extracted from freeze-dried mushrooms and substrates, essentially as described by Elmore et al. [50], with minor modifications. For the extraction, 300 mg of each sample were placed into 15 mL screw capped vials, 5 mL of hydrochloric acid 0.01 M was added and the samples were stirred for 15 min at room temperature on a magnetic stirrer plate. The extracts were allowed to settle for 45 min and then 1 mL aliquots of the supernatants were transferred to Eppendorf tubes and centrifuged at 7200× *g* for 30 min. The centrifuged supernatants were stored at −40 °C until AA analysis.

### 3.4. Crude Protein Content

Crude protein was calculated in freeze-dried substrate and mushroom samples on the basis of their nitrogen content which was determined by the Kjeldahl method using the converting factor ‘4.38’ for mushroom samples [51] and ‘6.25’ for cultivation substrates.

### 3.5. Derivatization of Free Amino Acids

FAAs profiling of the studied mushrooms and substrates was conducted using the EZ:faast™ amino acid derivatization technique for GC–MS (Phenomenex^®^, Torrance, CA, USA) [19]. The procedure consists of a solid phase extraction step followed by AAs derivatization and liquid/liquid extraction of the derivatives. According to the protocol, 100 μL aliquots of the centrifuged extracts were placed into the sample preparation vials provided, along with 20 nmol of norvaline as internal standard, followed by a solid phase extraction performed via sorbent tips that bind the AA derivatives, while allowing interfering compounds to flow through. The two-steps derivatization of the AAs was carried out at room temperature; the derivatized AAs were extracted into 100 μL of isooctane/chloroform, sealed in GC vials and analyzed immediately. 

### 3.6. GC/MS Analysis of Free Amino Acids

An Agilent GC 6890N gas chromatograph (Waldbronn, Germany), coupled with an HP5973 Mass Selective detector (Electron Impact, 70 eV), split–splitless injector and an HP7683 auto sampler was used for the analysis. Two μL of derivatized samples were injected into GC at a split ratio of 1:15. The separation was achieved using a Phenomenex Zebron ZB-A AAs analysis dedicated column (length = 10 m, internal diameter = 0.25 mm, film thickness = 25 µm). High purity helium was the carrier gas at a constant flow of 1.1 mL/min. The injector and transfer line temperatures were kept at 250 and 340 °C, respectively. The initial oven temperature was set at 110 °C, then increased to 320 °C at 30 °C/min where it was held for 3 min. A selective ion monitoring (SIM) GC–MS method was applied for the detection and quantification of 22 AAs, based on the ± 0.05 RT presence of target and qualifier ions at the predetermined ratios, together with the electronic library “Agilent.L” provided with the Ez:faast kit (Phenomenex^®^, Torrance, CA, USA). Quantification was carried out by employing norvaline as internal standard and constructing five points’ reference curves for each AA by standard AAs solutions provided with the kit. GABA, which was not present in the standards of the kit, was quantitated in the same way by employing pure GABA purchased from Sigma and by extracting the respective *m/z* ions (Supplementary Table S1). 

### 3.7. Statistical Analysis

All chemical analyses were performed in triplicate and data are presented as mean ± standard deviation. The Kolmogorov-Smirnov and Shapiro-Wilk tests were used to assess the normality of the data. As both tests provided *p* values >0.05 the null hypothesis, i.e., that the data are not normally distributed, was rejected and differences between means were established by conducting one-way ANOVA and Duncan’s t-test (5% level of probability).

In addition, PCA was performed on the entire AAs data set to attain an overview of possible interrelation between the AAs and the *Pleurotus* species examined. Statistical analysis was conducted with the SPSS software (SPSS for Windows, version 21.0, SPSS Inc., Chicago, IL, USA) and R-studio1.0.136/R3.3.3 loaded with the “ade4” and “adegraphics” packages.

## Figures and Tables

**Table 1:** Groups of amino acids studied. AA: amino acids; BCAA: branched-chain AAs; MSG: monosodium glutamate.

Essential AAs	BCAAs	MSG-Like AAs	Bitter Taste AAs	Sweet Taste AAs
Valine	Valine	Aspartic acid	Valine	Threonine
Leucine	Leucine	Glutamic acid	Methionine	Serine
Isoleucine	Isoleucine		Leucine	Glycine
Threonine			Isoleucine	Alanine
Methionine			Phenylalanine	Proline
Phenylalanine			Histidine	
Lysine			Tryptophan	
Histidine				
Tryptophan				

**Table 2:** Free amino acids and crude protein content in the mushroom cultivation substrates used.

Amino acid	Abbreviation	WS	GM	OL
Alanine	Ala	0.003 ± 0.001 a	0.065 ± 0.007 c	0.023 ± 0.005 b
Glycine	Gly	0.002 ± 0.001 a	0.022 ± 0.004 c	0.009 ± 0.000 b
Valine	Val	0.002 ± 0.001 a	0.010 ± 0.002 b	0.004 ± 0.000 a
Leucine	Leu	0.008 ± 0.005 a	0.032 ± 0.006 b	0.008 ± 0.001 a
Isoleucine	Ile	0.004 ± 0.002 a	0.019 ± 0.004 b	0.006 ± 0.001 a
Threonine	Thr	0.004 ± 0.002 a	0.020 ± 0.005 b	0.006 ± 0.000 a
Serine	Ser	0.005 ± 0.002 a	0.043 ± 0.003 c	0.012 ± 0.001 b
Proline	Pro	0.004 ± 0.001 a	0.091 ± 0.020 b	0.022 ± 0.001 a
Asparagine	Asn	0.003 ± 0.001 a	0.137 ± 0.002 c	0.061 ± 0.015 b
Thioproline	Thp	0.013 ± 0.005 a	0.105 ± 0.003 c	0.147 ± 0.004 b
Aspartic acid	Asp	0.012 ± 0.003 a	0.097 ± 0.006 c	0.138 ± 0.003 b
Methionine	Met	0.003 ± 0.002 a	0.001 ± 0.001 a	nd
Hydroxyproline	Hyp	nd	0.003 ± 0.001	nd
Glutamic acid	Glu	0.155 ± 0.026 a	0.204 ± 0.002 c	0.064 ± 0.002 b
Phenylalanine	Phe	0.006 ± 0.003 a	0.024 ± 0.005 b	0.006 ± 0.000 a
Glutamine	Gln	0.037 ± 0.010 a	0.093 ± 0.003 b	0.037 ± 0.004 a
Ornithine	Orn	0.022 ± 0.000 a	0.025 ± 0.001 b	0.022 ± 0.000 a
Lysine	Lys	0.033 ± 0.001 a	0.057 ± 0.006 b	0.035 ± 0.001 a
Histidine	His	0.0003 ± 0.000 a	0.023 ± 0.005 b	0.002 ± 0.000 a
Tyrosine	Tyr	0.006 ± 0.002 a	0.018 ± 0.003 b	0.008 ± 0.001 a
Tryptophan	Trp	0.001 ± 0.001 a	0.085 ± 0.001 b	0.093 ± 0.009 b
γ-Aminobutyric acid	GABA	nd	0.058 ± 0.003 a	0.008 ± 0.002 b
Sum of amino acids		0.319 ± 0.070	1.231 ± 0.077	0.711 ± 0.033
Crude protein		85.20 ± 4.34 a	116.60 ± 5.04 b	131.12 ± 0.09 c

Values (mg/g dry weight) represent the means ± SD (*n* = 3). Lack of letters in common indicates statistically significant differences (Duncan’s t-Test. *p* < 0.05) in comparisons of treatment means between different substrates. Abbreviations: WS, wheat straw; GM, wheat straw with grape marc (1:1 *w/w* ratio); OL, olive leaves with olive mill wastes (3:1 *w/w*); nd, not detected.

**Table 3:** Free amino acid and crude protein contents (mg/g dw) in mushrooms of *Pleurotus ostreatus*, *P. eryngii* and *P. nebrodensis* produced on three cultivation substrates.

Amino Acid	Substrate	*P. ostreatus*		*P. eryngii*		*P. nebrodensis*	
		LGAM 14	LGAM 11	UPA 12	LGAM 212	UPA 6	LGAM 162
Alanine	WS	7.22 ± 1.10 a	9.77 ± 2.29 a	4.74 ± 0.32 a	7.27 ± 1.08 a	2.82 ± 0.19 a	7.20 ± 1.47
	GM	10.34 ± 2.31 ab	13.33 ± 2.71 a	8.26 ± 1.11 b	8.54 ± 1.60 a	4.27 ± 0.69 a	7.53 ± 3.49
	OL	11.72 ± 2.69 b	10.42 ± 3.83 a	4.74 ± 1.99 a	7.61 ± 1.34 a	4.08 ± 1.04 a	*
Glycine	WS	1.03 ± 0.05 a	1.94 ± 0.97 a	1.03 ± 0.10 a	1.21 ± 0.56 a	0.29 ± 0.04 a	1.51 ± 0.17
	GM	1.85 ± 0.20 a	2.99 ± 0.35 a	1.88 ± 0.18 b	1.65 ± 0.11 a	0.73 ± 0.19 b	1.61 ± 0.55
	OL	3.29 ± 1.13 b	2.12 ± 0.82 a	0.98 ± 0.41 a	1.45 ± 0.10 a	0.50 ± 0.06 ab	*
Valine	WS	4.24 ± 0.34 a	6.94 ± 2.08 a	3.24 ± 0.12 a	4.81 ± 1.12 a	1.05 ± 0.14 a	3.97 ± 0.61
	GM	6.94 ± 0.84 ab	9.49 ± 1.05 a	5.26 ± 0.56 b	6.24 ± 0.53 a	1.76 ± 0.80 a	3.98 ± 1.54
	OL	8.44 ± 2.19 b	7.13 ± 2.01 a	3.35 ± 1.25 a	5.59 ± 0.83 a	1.56 ± 0.26 a	*
Leucine	WS	8.59 ± 1.32 a	12.24 ± 3.27 a	5.11 ± 0.50 a	8.88 ± 1.81 a	2.24 ± 0.15 a	8.29 ± 1.89
	GM	12.31 ± 2.50 a	16.45 ± 2.33 a	8.81 ± 1.11 a	11.24 ± 1.51 a	4.51 ± 1.22 b	7.81 ± 3.37
	OL	9.66 ± 8.19 a	9.95 ± 6.70 a	7.30 ± 3.07 a	10.10 ± 1.84 a	4.34 ± 1.10 b	*
Isoleucine	WS	2.80 ± 0.29 a	4.13 ± 0.96 a	2.15 ± 0.06 a	3.15 ± 0.77 a	0.67 ± 0.03 a	2.81 ± 0.53
	GM	4.35 ± 0.66 ab	5.79 ± 0.81 a	3.40 ± 0.33 a	4.13 ± 0.28 a	1.25 ± 0.54 a	2.70 ± 1.07
	OL	5.09 ± 1.21 b	4.38 ± 1.29 a	2.32 ± 0.92 a	3.62 ± 0.48 a	1.09 ± 0.24 a	*
Threonine	WS	2.29 ± 0.13 a	3.96 ± 1.56 a	1.93 ± 0.06 a	2.85 ± 0.87 a	0.61 ± 0.06 a	2.45 ± 0.31
	GM	3.83 ± 0.40 ab	5.65 ± 0.65 a	3.31 ± 0.26 b	3.68 ± 0.00 a	1.17 ± 0.44 a	2.47 ± 0.93
	OL	5.23 ± 1.27 b	4.19 ± 1.08 a	1.93 ± 0.77 a	3.22 ± 0.33 a	1.02 ± 0.20 a	*
Serine	WS	3.93 ± 0.14 a	6.17 ± 2.57 a	3.30 ± 0.24 a	4.81 ± 1.62 a	0.91 ± 0.21 a	4.17 ± 0.31
	GM	6.38 ± 0.50 b	8.81 ± 0.32 a	5.77 ± 0.64 b	5.97 ± 0.37 a	2.13 ± 0.60 b	4.19 ± 1.37
	OL	8.83 ± 1.29 c	6.95 ± 1.81 a	3.34 ± 1.29 a	5.11 ± 0.35 a	1.84 ± 0.24 b	*
Proline	WS	1.21 ± 0.06 a	2.30 ± 1.22 a	1.04 ± 0.09 a	1.22 ± 0.57 a	0.55 ± 0.06 a	1.37 ± 0.15
	GM	2.19 ± 0.25 a	3.77 ± 0.47 a	2.00 ± 0.26 b	1.67 ± 0.02 a	0.43 ± 0.20 a	1.35 ± 0.44
	OL	4.22 ± 1.59 b	2.69 ± 1.37 a	0.88 ± 0.31 a	1.57 ± 0.23 a	0.37 ± 0.06 a	*
Asparagine	WS	1.75 ± 0.10 a	2.82 ± 1.55 a	1.18 ± 0.06 a	1.77 ± 0.65 a	0.40 ± 0.05 a	1.63 ± 0.14
	GM	3.02 ± 0.35 b	4.52 ± 0.68 a	2.19 ± 0.23 b	2.26 ± 0.01 a	0.74 ± 0.24 b	1.57 ± 0.52
	OL	4.39 ± 1.03 c	3.25 ± 1.25 a	1.22 ± 0.49 a	1.95 ± 0.20 a	0.70 ± 0.10 ab	*
Thioproline	WS	2.48 ± 0.24 a	2.10 ± 0.38 a	0.82 ± 0.43 a	0.59 ± 0.44 a	0.71 ± 0.27 a	0.75 ± 0.11
	GM	3.57 ± 0.75 a	2.93 ± 0.93 a	0.93 ± 0.30 a	1.12 ± 0.03 a	2.69 ± 0.30 b	1.60 ± 0.80
	OL	3.31 ± 0.84 a	3.17 ± 2.14 a	1.17 ± 0.78 a	1.02 ± 0.78 a	1.97 ± 0.46 c	*
Aspartic acid	WS	2.51 ± 0.09 a	2.20 ± 0.03 a	0.98 ± 0.26 a	0.81 ± 0.57 a	0.76 ± 0.19 a	0.81 ± 0.10
	GM	3.51 ± 0.48 b	2.65 ± 0.80 a	1.14 ± 0.28 a	1.06 ± 0.12 a	2.59 ± 0.40 b	1.69 ± 0.62
	OL	3.55 ± 0.62 b	3.32 ± 2.12 a	1.22 ± 0.69 a	1.27 ± 0.85 a	1.94 ± 0.52 b	*
Methionine	WS	1.14 ± 0.12 a	1.86 ± 0.68 a	0.66 ± 0.21 a	1.36 ± 0.31 a	0.09 ± 0.01 a	1.31 ± 0.23
	GM	1.66 ± 0.21 ab	2.47 ± 0.14 a	1.08 ± 0.04 a	1.62 ± 0.08 a	0.58 ± 0.19 b	1.15 ± 0.42
	OL	2.10 ± 0.57 b	1.93 ± 0.42 a	0.90 ± 0.31 a	1.50 ± 0.23 a	0.51 ± 0.10 b	*
Hydroxyproline	WS	0.39 ± 0.04 a	0.48 ± 0.15 a	0.22 ± 0.08 a	0.40 ± 0.09 a	0.11 ± 0.10 a	0.34 ± 0.02
	GM	0.60 ± 0.09 b	0.71 ± 0.12 a	0.44 ± 0.08 b	0.44 ± 0.04 a	0.25 ± 0.08 a	0.36 ± 0.09
	OL	0.66 ± 0.11 b	0.58 ± 0.13 a	0.25 ± 0.07 a	0.38 ± 0.05 a	0.24 ± 0.01 a	*
Glutamic acid	WS	4.81 ± 0.92 a	5.68 ± 1.19 a	3.22 ± 0.48 a	4.92 ± 0.63 a	2.07 ± 0.09 a	4.42 ± 1.21
	GM	6.76 ± 2.08 a	7.84 ± 2.44 a	5.29 ± 0.70 b	5.73 ± 1.35 a	3.58 ± 0.62 a	4.59 ± 2.15
	OL	7.39 ± 2.32 a	6.76 ± 2.55 a	3.60 ± 1.47 ab	4.77 ± 0.81 a	3.46 ± 1.18 a	*
Phenylalanine	WS	2.95 ± 0.24 a	4.30 ± 1.51 a	1.71 ± 0.20 a	2.94 ± 0.71 a	0.97 ± 0.03 a	3.05 ± 0.54
	GM	4.14 ± 0.55 ab	6.01 ± 0.11 a	2.91 ± 0.19 b	3.57 ± 0.01 a	1.74 ± 0.41 b	2.81 ± 1.10
	OL	5.33 ± 1.16 b	4.68 ± 1.45 a	2.27 ± 0.83 ab	3.31 ± 0.57 a	1.98 ± 0.47 b	*
Glutamine	WS	7.80 ± 0.69 a	10.04 ± 6.97 a	3.35 ± 0.82 a	7.19 ± 0.87 a	0.84 ± 0.61 a	7.02 ± 0.69
	GM	14.08 ± 2.32 b	21.69 ± 6.93 a	8.28 ± 1.61 b	7.52 ± 1.04 a	4.22 ± 0.69 b	6.99 ± 1.17
	OL	20.03 ± 1.09 c	13.06 ± 8.46 a	4.66 ± 2.40 a	6.96 ± 1.74 a	6.42 ± 1.23 c	*
Ornithine	WS	0.65 ± 0.08 a	0.63 ± 0.24 a	0.65 ± 0.04 b	0.36 ± 0.08 a	0.42 ± 0.01 a	1.51 ± 0.29
	GM	0.93 ± 0.15 b	0.90 ± 0.25 a	0.82 ± 0.09 b	0.50 ± 0.19 a	0.76 ± 0.08 b	1.63 ± 0.67
	OL	1.10 ± 0.14 b	0.56 ± 0.28 a	0.31 ± 0.14 a	0.43 ± 0.09 a	0.32 ± 0.12 a	*
Lysine	WS	2.47 ± 0.27 a	3.23 ± 0.83 a	1.83 ± 0.09 a	2.77 ± 0.32 a	0.53 ± 0.03 a	2.79 ± 0.49
	GM	3.33 ± 0.78 a	4.29 ± 0.67 a	2.77 ± 0.41 a	3.42 ± 0.45 a	1.36 ± 0.44 b	2.61 ± 1.02
	OL	4.20 ± 1.30 a	3.59 ± 1.10 a	2.21 ± 1.02 a	2.96 ± 0.51 a	1.44 ± 0.30 b	*
Histidine	WS	0.88 ± 0.17 a	1.67 ± 1.24 a	0.52 ± 0.07 a	0.88 ± 0.50 a	0.10 ± 0.05 a	0.98 ± 0.21
	GM	1.63 ± 0.27 ab	2.59 ± 0.57 a	1.14 ± 0.26 b	1.14 ± 0.33 a	0.32 ± 0.21 a	0.86 ± 0.21
	OL	2.46 ± 1.03 b	1.66 ± 0.66 a	0.63 ± 0.20 a	1.04 ± 0.30 a	0.33 ± 0.06 a	*
Tyrosine	WS	2.66 ± 0.08 a	4.25 ± 1.73 a	1.54 ± 0.12 a	2.59 ± 0.62 a	0.95 ± 0.13 a	3.05 ± 0.46
	GM	3.99 ± 0.44 ab	5.93 ± 0.33 a	2.67 ± 0.24 b	3.18 ± 0.23 a	1.70 ± 0.42 b	2.81 ± 1.04
	OL	4.96 ± 1.08 b	4.34 ± 1.42 a	1.95 ± 0.70 ab	3.11 ± 0.54 a	1.40 ± 0.23 ab	*
Tryptophan	WS	1.07 ± 0.12 a	0.81 ± 0.71 a	0.59 ± 0.15 a	1.22 ± 0.68 a	0.18 ± 0.04 a	1.16 ± 0.34
	GM	1.80 ± 0.33 a	1.76 ± 0.00 a	1.14 ± 0.09 b	0.55 ± 0.78 a	0.43 ± 0.14 b	1.01 ± 0.33
	OL	1.70 ± 0.00 a	1.44 ± 0.22 a	0.76 ± 0.28 a	0.76 ± 0.66 a	0.42 ± 0.09 b	*
γ-Aminobutyric acid (GABA)	WS	0.21 ± 0.01 a	0.31 ± 0.20 a	0.08 ± 0.05 a	0.18 ± 0.13 a	0.09 ± 0.03 a	0.56 ± 0.26
	GM	0.43 ± 0.21 a	0.70 ± 0.41 a	0.38 ± 0.12 b	0.18 ± 0.03 a	0.18 ± 0.05 a	0.86 ± 0.37
	OL	0.46 ± 0.21 a	0.35 ± 0.21 a	0.06 ± 0.02 a	0.23 ± 0.08 a	0.36 ± 0.12 b	*
Total free amino acids	WS	63.10 ± 4.22 a	87.84 ± 29.53 a	62.19 ± 13.43 a	39.91 ± 1.58 a	17.37 ± 1.48 a	61.18 ± 9.42
	GM	97.03 ± 14.44 b	130.12 ± 17.16 a	75.44 ± 4.42 a	69.86 ± 5.81 b	37.42 ± 7.09 b	62.16 ± 22.76
	OL	116.98 ± 19.46 b	96.05 ± 31.79 a	67.89 ± 8.88 a	46.03 ± 19.17 a	36.30 ± 7.68 b	*
Crude protein	WS	164.07 ± 1.65 a	177.36 ± 3.55 a	154.63 ± 9.25 a	144.70 ± 2.90 b	121.10 ± 1.20 a	175.20 ± 5.26
	GM	221.18 ± 11.05 b	284.77 ± 2.85 b	187.83 ± 1.85 b	179.60 ± 5.40 c	175.23 ± 8.73 b	172.24 ± 10.33
	OL	293.20 ± 17.60 c	299.20 ± 9.00 c	167.23 ± 8.35 a	130.77 ± 7.84 a	130.50 ± 5.20 a	*

* Results are presented as means ± standard deviation (*n* = 3). Lack of letters in common indicates statistically significant differences (Duncan’s t-Test. *p* < 0.05) in comparisons of treatment means between different substrates. Abbreviations: dw, dry weight; WS, wheat straw; GM, wheat straw with grape marc (1:1 *w*/*w*); OL, olive leaves with olive mill wastes (3:1 *w*/*w* ratio); *, no mushroom production.

## Supplementary Materials

The following are available online, Supplementary Table S1: Retention times (Rt) and *m/z* of ions used for the selective ion monitoring of amino acids. Supplementary Table S2: Groups of free amino acids in *Pleurotus* mushrooms compared on the basis of the cultivation substrate used for each strain. Supplementary Figure S1: Score plot of PCA (PC1 vs. PC2) for the discrimination of *Pleurotus* cultivation substrates (GM, wheat straw with grape marc (1:1 *w/w*); OL, olive leaves with olive mill wastes (3:1 *w/w* ratio); WS, wheat straw).
