# Supplementary material for: Free Amino Acids in Three Pleurotus Species Cultivated on Agricultural and Agro-Industrial By-Products
Source: Molecules. 2020 Sep 2;25(17):4015. doi: 10.3390/molecules25174015 (PMC7504736; doi:10.3390/molecules25174015)
Supplement: Supplementary file 1 [file molecules-25-04015-s001.pdf]

molecules

Supplementary material

Table S1. Retention times (Rt) and m/z of ions used for the selective ion monitoring of amino acids.

Target Compounds	Abbreviation	Rt (min)	Ions (m/z)*
Alanine	Ala	1.063	130, 88
Glycine	Gly	1.163	116, 207
Valine	Val	1.357	158, 116
Norvaline (internal standard)	Nva	1.478	158,72
Leucine	Leu	1.623	172, 86
Isoleucine	Ile	1.622	172, 130
Threonine	Thr	1.841	160, 101
γ-Aminobutyric acid	GABA	1.850	130, 86, 112, 172
Serine	Ser	1.866	146, 203
Proline	Pro	1.935	156, 243
Asparagine	Asn	2.038	155, 69
Thioproline	Thp	2.590	174, 147
Aspartic acid	Asp	2.597	216, 130
Methionine	Met	2.619	203, 277
4-Hydroxyproline	4Hyp	2.969	172, 86, 130
Glutamic acid	Glu	2.971	230, 170
Phenylalanine	Phe	2.982	206, 190
Glutamine	Gln	3.614	84, 187
Ornithine	Orn	4.019	156, 70
Lysine	Lys	4.292	170, 128
Histidine	His	4.468	282, 168
Tyrosine	Tyr	4.759	206, 107
Tryptophan	Trp	5.028	130
Cystine	Cys	5.763	248, 216

*: User's Manual; Phenomenex® EZ:faast™ Free (Physiological) Amino Acid Analysis by GC-MS for Agilent GC/MS instruments.

Table **S2.** Groups of free amino acids in *Pleurotus* mushrooms compared on the basis of the cultivation substrate used for each strain.

AA	Substrates	*P. ostreatus*		*P. eryngii*		*P. nebrodensis*	
		LGAM 11	LGAM 14	LGAM 212	UPA 12	UPA 6	LGAM 162
Essential AAs	WS	26.44±2.33a	39.13±11.19a	28.87±6.76a	17.75±1.13a	6.43±0.05a	26.82±4.62
	GM	39.39±5.30b	53.33±5.49a	35.60±3.12a	29.81±2.34b	13.13±4.33b	25.40±9.86
	OL	43.08±9.53b	38.49±12.69a	32.10±4.18a	21.67±8.61ab	12.69±2.73b	*
BCAAs	WS	15.64±1.90a	23.30±6.26a	16.83±3.64a	10.51±0.54a	3.96±0.04a	15.07±3.01
	GM	23.59±3.94a	31.73±4.04a	21.61±2.31a	17.47±1.90b	7.52±2.51b	14.49±5.97
	OL	23.19±7.78a	21.46±8.99a	19.31±3.13a	12.97±5.24ab	6.98±1.58ab	*
MSG-like AAs	WS	7.32±1.01a	7.89±1.17a	5.73±0.88a	4.20±0.73a	2.83±0.26a	5.24±1.23
	GM	10.27±2.53a	10.49±1.98a	6.79±1.23a	6.43±0.81a	6.18±1.00b	6.28±2.75
	OL	10.94±2.85a	10.07±4.30a	6.04±0.97a	4.82±2.17a	5.40±1.71b	*
Bitter AAs	WS	21.68±1.96a	31.95±8.84a	23.25±5.67a	13.99±1.16a	5.29±0.01a	21.57±3.87
	GM	32.22±4.14ab	43.39±4.51a	28.50±2.67a	23.74±1.82b	10.59±3.44b	20.31±7.92
	OL	33.65±8.30b	30.71±10.87a	25.92±3.43a	17.53±6.85ab	10.23±2.23b	*
Sweet AAs	WS	15.68±1.18a	24.15±8.31a	17.37±4.31a	12.04±0.49a	5.19±0.23a	16.70±2.20
	GM	24.59±3.50ab	34.56±3.51a	21.52±1.10a	21.22±1.42b	8.74±1.89b	17.14±6.73
	OL	33.28±7.07b	26.37±8.57a	18.96±2.20a	11.88±4.77a	7.82±1.48ab	*

Values (mg/g dry weight), are expressed as means ± standard deviation (n=3); Lack of letter in common denotes statistically significant differences among means by Duncan's multiple comparison test at $p < 0.05$, in comparison of species in each substrate; WS, wheat straw; GM, wheat straw with grape marc (1:1 w/w); OL, olive leaves with olive mill wastes (3:1 w/w ratio); * No mushroom production; Essential amino acids, Thr + Val + Met + Ile + Leu + Phe + Lys + His+ Trp; BCAAs Branched chain amino acids, Val + Ile + Leu; MSG-like, monosodium glutamate-like, Asp + Glu; Bitter, Val + Met + Ile + Leu + Phe + His + Trp; Sweet, Thr + Ser + Gly + Ala + Pro.

Figure S1. Score plot of PCA (PC1 vs. PC2) for the discrimination of *Pleurotus* cultivation substrates.

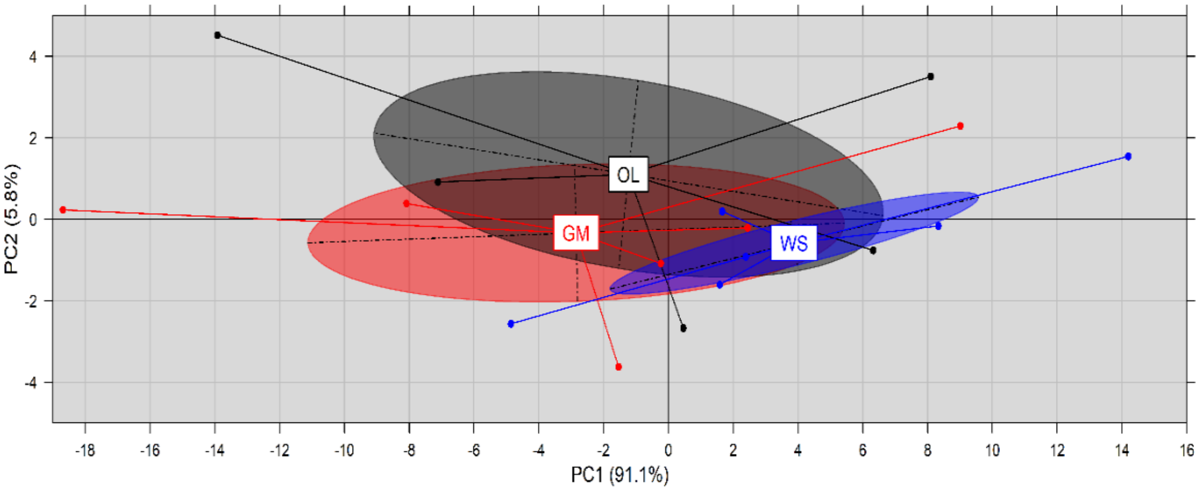

(GM, wheat straw with grape marc (1:1 w/w); OL, olive leaves with olive mill wastes (3:1 w/w ratio); WS, wheat straw).
